# Factors associated with dyslipidemia and its prevalence among Awash wine factory employees, Addis Ababa, Ethiopia: a cross-sectional study

**DOI:** 10.1186/s12872-022-02465-4

**Published:** 2022-01-30

**Authors:** Daniel Angassa, Samrawit Solomon, Awol Seid

**Affiliations:** 1 Lideta Sub-City Health Office, Addis Ababa, Ethiopia; 2 School of Public Health, St. Paul’s Hospital Millennium Medical College, Addis Ababa, Ethiopia

**Keywords:** Dyslipidemias, Prevalence, Risk factors, Wine factory, Ethiopia

## Abstract

**Background:**

Dyslipidemia is a highly prevalent and modifiable risk factor for atherosclerotic cardiovascular diseases. Though the problem is significant in Ethiopia, available data in this regard is very poor among alcoholic beverage industrial workers. This study aimed to assess factors associated with dyslipidemia and its prevalence among Awash wine factory employees in Addis Ababa, Ethiopia.

**Methods:**

A cross-sectional study was conducted among 335 randomly selected employees of Awash wine factory, Addis Ababa, Ethiopia from January to February 2021. Data was collected by a face-to-face interview technique using the WHO STEPwise approach. Data were entered and analyzed using Epi Info 7 and SPSS version 26, respectively. Both bivariable and multivariable logistic regression analyses were performed to identify factors associated with dyslipidemia. All statistical tests were declared significant at *p*-value < 0.05.

**Results:**

The overall prevalence of dyslipidemia was 67.8% (95% CI 62.5–72.7%). Elevated total cholesterol, elevated triglycerides, reduced high-density lipoprotein, and elevated low-density lipoprotein was found in 25.4%, 33.4%, 50.7%, and 21.5% of participants, respectively. Dyslipidemia was significantly associated with age group 30–39 years (AOR = 2.51; 95% CI 1.16–5.44, *p* = 0.019), ≥ 40 years (AOR = 6.45; 95% CI 2.01–20.71, *p* = 0.002), current alcohol consumption (AOR = 3.37; 95% CI 1.70–6.66, *p* < 0.001), eating vegetables < 2 days per week (AOR = 2.89; 95% CI 1.54–5.43, *p* = 0.001), sitting duration of > 4 h per day (AOR = 1.96; 95% CI 1.03–3.74, *p* = 0.041), and raised waist circumference (AOR = 4.56; 95% CI 2.07–10.08, *p* < 0.001).

**Conclusions:**

High prevalence of dyslipidemia was found among Awash wine factory employees in Addis Ababa. Periodic screening of high-risk groups along with effective health promotion and education which encourages a healthy lifestyle is essential.

## Background

Globally, cardiovascular diseases (CVD) are the leading cause of mortality with high incidence and prevalence in countries of all economic groups. In 2016, CVD were responsible for 17.9 million deaths worldwide, accounting for 31% of all global deaths [1–3]. In recent decades, most African countries including Ethiopia have been undergoing an epidemiological transition which manifested by the emergence of cardiovascular diseases. Among the modifiable CVD risk factors, dyslipidemia is the commonest risk factor globally and even in Africa. At least one in five adults in the general population of Africa has dyslipidemia [4, 5].

Dyslipidemia is a condition that occurs because of abnormalities in the plasma lipids such as elevated plasma total cholesterol (TC), elevated low-density lipoprotein cholesterol (LDL-C), elevated triglycerides (TG), and reduced high-density lipoprotein cholesterol (HDL-C) levels, occurring singly or in combinations [6]. It is a highly prevalent and modifiable risk factor for atherosclerotic cardiovascular diseases (CVD) such as coronary heart diseases (CHD) resulting in serious morbidity, mortality, and medical costs worldwide [7].

In 2016, the Global Burden of Disease study reported that high concentrations of total cholesterol caused about 4·4 million deaths and 93·8 million disability-adjusted life-years (DALYs) [8]. In Africa, a meta-analysis conducted in 2018 showed an overall 29.7% prevalence of dyslipidemia with a higher prevalence in urban areas. In sub-Saharan Africa, a high prevalence of more than 35% is reported in some areas [5]. A national survey conducted in Ethiopia in 2015 showed a 68% and 21% prevalence of low HDL-cholesterol and hypertriglyceridemia, respectively [9]. Previous observational studies indicated that excessive alcohol intake largely contributes to the development of dyslipidemia mainly hypertriglyceridemia. A dose–response relationship has been also described between alcohol intake and blood lipids, especially with HDL-C, LDL-C, and triglyceride (TG) levels [10–12].

Furthermore, recent studies showed that using PCSK9 inhibitors had a multimodal benefit in improving the quality of life in patients at high cardiovascular risk. Besides providing a good clinical prognostic improvement mainly reducing LDL-C levels substantially, it had also an economic advantage due to its cost-effectiveness [13]. The additional benefit of the use of PCSK9 inhibitors in high cardiovascular risk patients is significantly higher adherence and low discontinuation rate than other lipid-lowering drugs [14].

Even if, various risk factors of dyslipidemia have been identified through numerous studies, most of the studies focused on participants with co-morbidities such as diabetes mellitus, hypertension, and HIV/AIDS [15–17]. And despite its association with heavy alcohol consumption [10, 11], available data regarding the prevalence of dyslipidemia in alcoholic beverage industrial workplaces in Ethiopia is very poor. Therefore, this study aimed to assess factors associated with dyslipidemia and its prevalence among Awash wine factory employees in Addis Ababa, Ethiopia.

## Methods

### Study design and setting

An institution-based cross-sectional study design was conducted from January to February 2021 in Awash Wine factory which was selected purposively based on the availability of recent laboratory data of its employees. Awash wine factory is one of the many alcoholic beverage factories in Ethiopia which produces wine. It is the oldest wine factory in the country, established in 1943. It has three sites in Addis Ababa with a total of 653 permanent employees. All permanent employees 18 years of age and above and who have been working at the wine factory for at least one year were included in this study. Whereas, pregnant women, employees with a medical report older than November 2020, and incomplete medical files were excluded.

### Sample size and sampling technique

The required sample size was determined by using a single population proportion formula based on a similar study conducted among adult residents of Mekelle city, northern Ethiopia which showed an estimated 66.7% prevalence of dyslipidemia [18] with the assumption of 95% level of confidence, 4% margin of error, and 80% power. After using a finite population correction formula and considering a 10% non-response rate, the calculated sample size was 322.

A simple random sampling technique was used to select the study participants from all three sites of the factory. First, the total number of permanent employees from all three sites in Addis Ababa was identified. Then, after obtaining a list of all permanent employees of each site from the human resource department of the factory, participants were selected using a simple random sampling method after assigning study identification numbers to each employee on the sampling frame to minimize selection bias.

### Data collection

Data was collected by a face-to-face interview technique using a standardized questionnaire adopted from the WHO STEPwise approach for chronic disease risk factors [19] after performing some modifications. First, the modified questionnaire which is in the English language was translated into the local language which is Amharic and translated back to English by another person to check for its consistency. The data collectors were health care professionals (two nurses and one health officer) working in the clinics of the factory. One data collector was selected from each site and two days of training on the data collection process was conducted. After obtaining informed consent, data collectors administered the questionnaire using face-to-face interviews and performed physical measurements using standardized instruments. Before the actual data collection started, the adopted questionnaire was pre-tested on 5% (17) of the sample size among employees of a similar alcohol factory, National Alcohol and Liquor Factory, for its consistency and sensitivity.

Body weight was measured in kilograms without shoes and in light clothes using a calibrated weight scale. Body height was also measured in centimeters without shoes in standing upright position on a flat surface. Body Mass Index (BMI) was calculated as weight in kg divided by height in meters squared. Waist circumference was measured by using a non-elastic tape around the bare abdomen at the midpoint between the lower margin of the last palpable rib and the top of the iliac crest of the hip bone. Blood pressure was measured using a standard mercury sphygmomanometer on the right arm in a sitting position after participants rested for a minimum of 10 min. Blood pressure was measured twice 10 min apart and the average reading was recorded.

Fasting blood glucose levels and all the lipid profiles (total cholesterol, triglyceride, LDL, and HDL cholesterols) were obtained from the medical records of the employees of the factory which was conducted in November 2020. These medical records are a result of the annual medical examination of all permanent employees of the factory as part of the plan of the health care department of the factory.

### Definitions

Dyslipidemia is defined based on the National Cholesterol Education Program-Adult Treatment Panel III (NCEP-ATP III) guidelines as a total cholesterol level ≥ 200 mg/dl, triglycerides ≥ 150 mg/dl, LDL cholesterol ≥ 130 mg/dl, and HDL cholesterol < 40 mg/dl for men and < 50 mg/dl for women [20]. Abnormal levels of any of the above lipid parameters were considered as dyslipidemia.

International diabetic federation (IDF) defined raised fasting blood glucose as (FBG) level ≥ 100 mg/dl or previously diagnosed diabetes or receiving treatment for diabetes. Raised blood pressure is defined as systolic blood pressure (SBP) ≥ 130 mmHg and/or diastolic blood pressure (DBP) ≥ 85 mmHg or on the treatment of previously diagnosed hypertension. Body mass index (BMI) was used to classify underweight: BMI < 18.5 kg/m^2^, normal: BMI: 18.5–24.9 kg/m^2^, overweight: BMI: ≥ 25 kg/m^2^, and obese: BMI ≥ 30 kg/m^2^. Abdominal obesity is defined as waist circumference ≥ 94 cm for men and ≥ 80 cm for women [21].

Heavy Alcohol Consumption is defined as the average consumption of 5 or more standard alcoholic drinks per day for men (≈50gm of alcohol) or 4 or more alcoholic drinks (or 40gm alcohol) for women. A standard alcoholic drink is the equivalent of one glass/can/bottle (330 ml) of regular beer (with 3% ethanol), one glass (100 ml) of wine (10% ethanol), or one glass or measure (40 ml) of distilled spirit, each of which adds up to about 10 g of ethanol per drink.

Regarding physical activity, vigorous-intensity activities are activities that require hard physical effort and cause large increases in breathing or heart rate such as heavy lifting, aerobic, or fast bicycling. Moderate-intensity activities are activities that cause small increases in breathing or heart rate such as carrying light loads, bicycling at a regular pace, or doubles tennis. Walking: this includes walking to travel from place to place, and any other walking that you might do solely for recreation, sport, exercise, or leisure. And Sitting: include time spent in class, at home, while doing course work, and during leisure time [19].

### Data processing and analysis

The collected data were entered into Epi Info version 7 and exported to SPSS version 26 statistical software for analysis. Data cleaning was performed to check for frequencies, missed or error values, and identified errors were corrected after revision of the original completed questionnaire. Descriptive statistics such as frequency, proportion, mean and standard deviation (SD) were used to summarize variables and evaluate the distribution of responses. Both bivariable and multivariable logistic regression analyses were performed to identify factors associated with dyslipidemia. Variables that were significant at *p*-value < 0.05 in the bivariable analysis were included in the multivariable analysis to control confounding effects. Adjusted odds ratios (AOR) with 95% confidence intervals (95% CI) were used to determine the strength of associations between dyslipidemia and independent variables and the level of statistical significance was declared at *p*-value < 0.05. The goodness of fit of the model was checked using the Hosmer–Lemeshow test at *p* > 0.05.

## Results

### Socio-demographic and anthropometric characteristics of the participants

A total of 335 participants were included in this study making the response rate 100%. The majority of participants (63.6%) were male and 36.4% were female. About one-third (33.1%) of the participants were aged 40 years and above, while 43.6% were in the age group of 30–39 years with the mean age ± SD of 36.9 ± 8.9 years (ranging from 21 to 62 years). Regarding their educational level, all participants have attained education from primary to higher education levels and about half (53.1%) of them have completed college or university level education. More than half (55.2%) of the participants were married and about two-third (67.5%) were working in the plant/factory department. About a quarter (26%) of the participants have worked in the factory for 5–9 years and about half (51%) were earning 5000 to 10,000 ETB monthly (Table 1).

**Table 1:** Distribution of dyslipidemia by socio-demographic and anthropometric characteristics of employees of Awash wine factory in Addis Ababa, Ethiopia, 2021 (n = 335)

Variables		Dyslipidemia	
	Total	Yes	No
	N (%)	n (%)	n (%)
*Age* (*years*)			
< 30	78 (23.3)	30 (38.5)	48 (61.5)
30–39	146 (43.6)	100 (68.5)	46 (31.5)
> = 40	111 (33.1)	97 (87.4)	14 (12.6)
*Gender*			
Male	213 (63.6)	140 (65.7)	73 (34.3)
Female	122 (36.4)	87 (71.3)	35 (28.7)
*Educational* *level*			
Primary School	89 (26.6)	57 (64.0)	32 (36.0)
Secondary School	68 (20.3)	48 (70.6)	20 (29.4)
College/University	178 (53.1)	122 (68.5)	56 (31.5)
*Marital* *Status*			
Unmarried^a^	150 (44.8)	85 (56.7)	65 (43.3)
Married	185 (55.2)	142 (76.8)	43 (23.2)
*Working* *department*			
Plant (factory)	226 (67.5)	157 (69.5)	69 (30.5)
Office	86 (25.7)	54 (62.8)	32 (37.2)
Others^b^	23 (6.9)	16 (69.6)	7 (30.4)
*Total* *service* *years*			
< 5	132 (39.4)	70 (53.0)	62 (47.0)
5–9	87 (26.0)	58 (66.7)	29 (33.3)
≥ 10	116 (34.6)	99 (85.3)	17 (14.7)
*Monthly* *income* (*ETB*)			
< 5000	76 (22.7)	41 (53.9)	35 (46.1)
5000–9999	171 (51.0)	124 (72.5)	47 (27.5)
≥ 10,000	88 (26.3)	62 (70.5	26 (29.5)
*Waist* *circumference* (*cm*) (*Male/Female*)			
< 94/80	180 (53.7)	93 (51.7)	87 (48.3)
≥ 94/80	155 (46.3)	134 (86.5)	21 (13.5)
*Body* *Mass* *Index* (*kg/m*^2^)			
< 18.5	5 (1.5)	2 (40.0)	3 (60.0)
18.5–24.9	206 (61.5)	118 (57.3)	88 (42.7)
25–29.9	94 (28.1)	80 (85.1)	14 (14.9)
≥ 30	30 (9.0)	27 (90.0)	3 (10.0)
*Blood* *pressure*			
Normal^c^	252 (75.2)	156 (61.9)	96 (38.1)
Raised^d^	83 (24.8)	71 (85.5)	12 (14.5)
*Fasting* *blood* *glucose* (*mg/dl*)			
< 100	297 (88.7)	194 (65.3)	103 (34.7)
≥ 100	38 (11.3)	33 (86.8)	5 (13.2)

^a^Single, divorced, separated, widowed

^b^Drivers and salesmen

^c^Blood pressure < 130/85 mmHg

^d^Blood pressure ≥ 130/85 mmHg

Of the total participants, one hundred and fifty-one (45.1%) had raised waist circumference. More than a quarter (28.1%) of the participants were overweight, while 30 (9%) were obese. Eighty-three (24.8%) participants had raised blood pressure. However, only 16 (19.3%) of them were on anti-hypertensive medication and 13% were not aware of their elevated blood pressure status. Thirty-eight (11.3%) participants had raised fasting blood glucose levels and less than half of them (44.7%) were on anti-diabetic medication (Table 1).

### Behavioral and lifestyle characteristics of the participants

Of the total study participants, current tobacco and chat use were reported by 23 (6.9%) and 47 (14%), respectively. Nearly three-quarters (74.3%) of the participants consumed alcohol over the past year preceding the time of data collection. Among current alcohol consumers, 23 (9.2%) were daily consumers, and 70 (28.1%) consumed 2–3 days per week. Two-thirds (65.4%) of the participants eat vegetables fewer than two days per week, and the majority (82.1%) eat fruits fewer than two days per week. Nearly a third (33.1%) of the participants eat eggs for two days or more per week. Thirty-nine (11.6%) of the participants were engaged in vigorous-intensity physical activities, while 107 (31.9%) reported regular walking for 30 min or more. More than half (54.6%) of the participants reported a sitting duration of fewer than four hours per day, and the majority (88.4%) reported six or more hours of sleeping duration per day (Table 2).

**Table 2:** Distribution of dyslipidemia by behavioral and lifestyle characteristics of employees of Awash wine factory in Addis Ababa, Ethiopia, 2021 (n = 335)

Variables		Dyslipidemia	
	Total	Yes	No
	N (%)	n (%)	n (%)
*Current* *tobacco* *smoking*			
No	312 (93.1)	209 (67.0)	103 (33.0)
Yes	23 (6.3)	18 (78.3)	5 (21.7)
*Current* *alcohol* *consumption*			
No	86 (25.7)	47 (54.7)	39 (45.3)
Yes	249 (74.3)	180 (72.3)	69 (27.7)
*Current* *chat* *chewing*			
No	288 (86.0)	193 (67.0)	95 (33.0)
Yes	47 (14.0)	34 (72.3)	13 (27.7)
*Vegetables* *eating* *frequency*			
≥ 2 days per week	116 (34.6)	66 (56.9)	50 (43.1)
< 2 days per week	219 (65.4)	161 (73.5)	58 (26.5)
*Fruits* *eating* *frequency*			
≥ 2 days per week	60 (17.9)	35 (58.3)	25 (41.7)
< 2 days per week	275 (82.1)	192 (69.8)	83 (30.2)
*Eggs* *eating* *frequency*			
≥ 2 days per week	111 (33.1)	68 (61.3)	43 (38.7)
< 2 days per week	224 (66.9)	159 (71.0)	65 (29.0)
*Vigorous-intensity* *physical* *activities*			
No	296 (88.4)	206 (69.6)	90 (30.4)
Yes	39 (11.6)	21 (53.8)	18 (46.2)
*Regular* *walking* *for* *30* *min* *or* *more*			
No	228 (68.1)	71 (66.4)	72 (31.6)
Yes	107 (31.9)	156 (68.4)	36 (33.6)
*Sitting* *duration* *per* *day* (*hours*)			
< 4	183 (54.6)	114 (62.3)	69 (37.7)
≥ 4	152 (45.4)	113 (74.3)	39 (25.7)
*Sleeping* *duration* *per* *day* (*hours*)			
< 6	39 (11.6)	25 (64.1)	14 (35.9)
≥ 6	296 (88.4)	202 (68.2)	94 (31.8)

### Prevalence of dyslipidemia

**Fig. 1:**
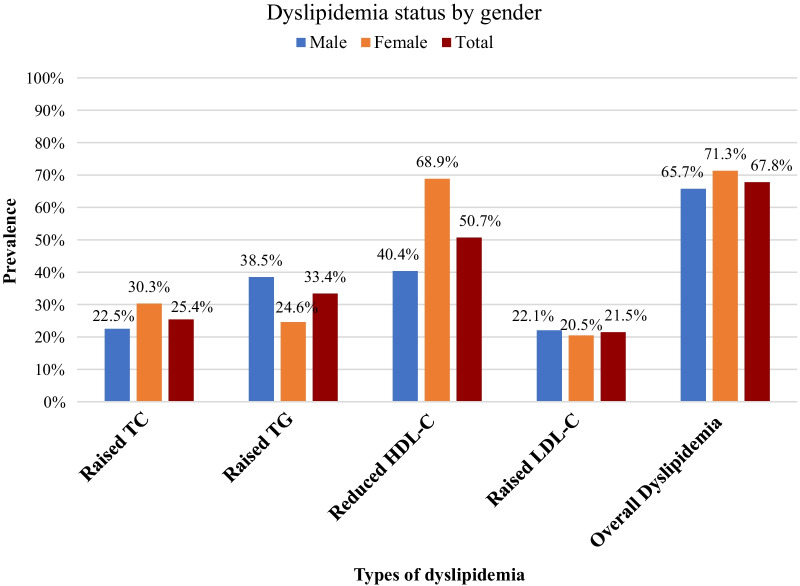
Prevalence of dyslipidemia by gender among employees of Awash wine factory in Addis Ababa, Ethiopia, 2021 (n = 335)

The overall prevalence of dyslipidemia in this study was 67.8% (95% CI 62.5–72.7%). Among individual lipid abnormalities, the highest prevalence was for reduced HDL-C level (50.7%), followed by elevated triglycerides (33.4%), elevated total cholesterol (25.4%), and elevated LDL-C (21.5%). The mean values of total cholesterol, triglycerides, HDL-C, and LDL-C were 177.5 ± 42.6 mg/dl, 145.9 ± 100 mg/dl, 43.2 ± 8.3 mg/dl, and 106.3 ± 30.7 mg/dl, respectively. The prevalence of reduced HDL-C level was higher among women (68.9%) and elevated triglyceride level was higher among men (38.5%) (Fig. 1).

### Factors associated with dyslipidemia

Eleven independent variables that were significant at *p*-value < 0.05 in the bivariable logistic regression analysis were entered into the multivariable logistic regression analysis to identify factors independently associated with dyslipidemia. According to the multivariable logistic regression analysis, older age, alcohol consumption, eating vegetables for two days or less per week, sitting four hours or more per day, and raised waist circumference had a statistically significant association with the prevalence of dyslipidemia. Participants aged 30–39 years and 40 years and older were about two times (AOR = 2.51; 95% CI 1.16–5.44, *p* = 0.019) and six times (AOR = 6.45; 95% CI 2.01–20.71, *p* = 0.002) more likely to develop dyslipidemia, respectively, as compared to those less than 30 years of age. Current alcohol consumers were about three times more likely to develop dyslipidemia (AOR = 3.37; 95% CI 1.70–6.66, *p* < 0.001) as compared to those who don't consume alcohol. Participants who eat vegetables fewer than two days per week had a 2.9 (AOR = 2.89; 95% CI 1.54–5.43, *p* = 0.001) times higher risk of developing dyslipidemia when compared to their counterparts. Similarly, participants who sit four hours or more per day were about two times more likely to develop dyslipidemia (AOR = 1.96; 95% CI 1.03–3.74, *p* = 0.041) as compared to those with a sitting duration of fewer than four hours per day. The odds of dyslipidemia among participants with raised waist circumference was 4.6 (AOR = 4.56; 95% CI 2.07–10.08, *p* < 0.001) times higher as compared to those with a normal waist circumference (Table 3).

**Table 3:** Multivariable logistic regression analysis of factors associated with dyslipidemia among employees of Awash wine factory in Addis Ababa, Ethiopia, 2021 (n = 335)

Variables	Dyslipidemia				AOR (95% CI)	*p*-value
	Yes	No	COR (95% CI)	*p*-value		
	n (%)	n (%)				
*Age* (*years*)						
< 30	30 (38.5)	48 (61.5)	1		1	
30–39	100 (68.5)	46 (31.5)	3.48 (1.96–6.18)	< 0.001	2.51 (1.16–5.44)	0.019
≥ 40	97 (87.4)	14 (12.6)	11.09 (5.38–22.83)	< 0.001	6.45 (2.01–20.71)	0.002
*Marital* *status*						
Unmarried^a^	85 (56.7)	65 (43.3)	1		1	
Married	142 (76.8)	43 (23.2)	2.52 (1.58–4.04)	< 0.001	1.43 (0.73–2.82)	0.300
*Total* *service* *years*						
< 5	70 (53.0)	62 (47.0)	1		1	
5–9	58 (66.7)	29 (33.3)	1.77 (1.01–3.11)	0.046	1.39 (0.65–2.99)	0.395
≥ 10	99 (85.3)	17 (14.7)	5.16 (2.78–9.57)	< 0.001	1.83 (0.71–4.70)	0.208
*Monthly* *income* (*ETB*)						
< 5000	41 (53.9)	35 (46.1)	1		1	
5000–9999	124 (72.5)	47 (27.5)	2.52 (1.28–3.95)	0.005	1.83 (0.88–3.80)	0.104
≥ 10,000	62 (70.5)	26 (29.5)	2.04 (1.07–3.87)	0.030	1.11 (0.45–2.72)	0.827
*Current* *alcohol* *consumption*						
No	47 (54.7)	39 (45.3)	1		1	
Yes	180 (72.3)	69 (27.7)	2.17 (1.30–3.59)	0.003	3.37 (1.70–6.66)	< 0.001
*Vegetables* *eating* *frequency*						
≥ 2 days per week	66 (56.9)	50 (43.1)	1		1	
< 2 days per week	161 (73.5)	58 (26.5)	2.10 (1.31–3.38)	0.002	2.89 (1.54–5.43)	0.001
*Sitting* *duration* *per* *day* (*hours*)						
< 4	121 (63.7)	69 (36.3)	1		1	
≥ 4	106 (73.1)	39 (26.9)	1.75 (1.09–2.81)	0.019	1.96 (1.03–3.74)	0.041
*Waist* *circumference* (*cm*) (*Male/Female*)						
< 94/80	97 (52.7)	87 (47.3)	1		1	
≥ 94/80	130 (86.1)	21 (13.9)	5.97 (3.46–10.29)	< 0.001	4.56 (2.07–10.08)	< 0.001
*Body* *Mass* *Index* (*kg/m*^2^)						
< 18.5	2 (40.0)	3 (60.0)	1		1	
18.5–24.9	118 (57.3)	88 (42.7)	2.01 (0.33–12.29)	0.449	1.12 (0.13–9.61)	0.919
25–29.9	80 (85.1)	14 (14.9)	8.57 (1.31–56.01)	0.025	1.64 (0.17–16.12)	0.673
≥ 30	27 (90.0)	3 (10.0)	13.50 (1.57–115.93)	0.018	2.58 (0.19–33.51)	0.468
*Blood* *pressure*						
Normal^b^	156 (61.9)	96 (38.1)	1		1	
Raised^c^	71 (85.5)	12 (14.5)	3.64 (1.88–7.06)	< 0.001	0.56 (0.21–1.47)	0.240
*Fasting* *blood* *glucose* (*mg/dl*)						
< 100	194 (65.3)	103 (34.7)	1		1	
≥ 100	33 (86.8)	5 (13.2)	3.50 (1.33–9.25)	0.011	2.76 (0.83–9.21)	0.098

COR, Crude Odds Ratio; AOR, Adjusted Odds Ratio; CI, Confidence Interval

^a^Single, divorced, separated, widowed

^b^Blood pressure < 130/85 mmHg

^c^ Blood pressure ≥ 130/85 mmHg

## Discussion

Dyslipidemia with its increasing prevalence is a common public health problem in developing countries and a major contributing factor to the development of cardiovascular diseases. This study was conducted to assess factors associated with dyslipidemia and its prevalence among Awash wine factory employees in Addis Ababa, Ethiopia. Our findings from this study revealed a high prevalence of dyslipidemia, 67.8% (95% CI 62.5–72.7%) among employees of Awash wine factory in Addis Ababa. Reduced HDL-C was the most prevalent type of dyslipidemia, 50.7%, followed by elevated triglycerides, 33.4%. The prevalence of elevated total cholesterol and elevated LDL-C was 25.4% and 21.5%, respectively. Participants with older age and raised waist circumference had a higher risk of developing dyslipidemia. Moreover, consuming alcohol, eating vegetables for two days or less per week, and sitting four hours or more per day were significantly associated with dyslipidemia.

The overall prevalence of dyslipidemia reported in this study is consistent with the findings reported in Mekelle, 66.7% [18], and Durame, southern Ethiopia, 65.6% [15]. However, the prevalence is higher than the findings reported in Africa, 29.7% [4], Eastern Ethiopia, 34.8% [6], China, 43.3% [1], India, 50.7% [22], Iran, 51.8% [23], Sao Paulo, 59.74% [24], Nigeria, 60% [25], and Togo, 60.3% [5]. The reason for the high prevalence in this study might be attributed to differences in sample sizes, study setting, study population, socio-economic status, and lifestyle of the study participants [23]. In contrast, the prevalence is lower than the findings reported in Iran, 83.4% [26], and Hawassa, southern Ethiopia, 90.8% [16]. The presence of co-morbidity might be the main reason for the higher prevalence in the study from Southern Ethiopia [20], as it was conducted among hypertensive patients.

The most prevalent type of dyslipidemia was reduced HDL-C, 50.7% followed by elevated triglycerides, 33.4%, which is comparable with the findings from Ethiopia, [9], Iran [23], and Venezuela [27]. The prevalence of reduced HDL-C in this study, 50.7% is higher than the findings reported in Togo, 16.4% [5], Mekelle, 16.5% [18], China, 20.8% [1], and Durame, southern Ethiopia, 41.9% [15]. Whereas, it is lower than the findings from India, 62% [22], and Ethiopia, 68.7% [9]. These variations might be due to differences in cut-off values, distribution of risk factors, study setting, and socio-economic status of the participants.

The prevalence of elevated triglyceride in this study, 33.4% is higher than the findings reported in Nigeria, 5% [25], Africa, 17% [4], Ethiopia, 21% [9], China, 22.5% [1], and India, 27% [22]. However, higher prevalence were reported in studies conducted in Venezuela, 39.7% [27], Mekelle, 40.2% [18], Saudi Arabia, 44% [7], and Hawassa, southern Ethiopia, 62.2% [16].

The prevalence of elevated total cholesterol in this study, 25.4% is almost consistent with the study findings reported in Venezuela, 22.2% [27], Nigeria, 23% [25], Durame, southern Ethiopia, 23.7% [15], and Africa, 25.5% [4]. Whereas, higher prevalence of elevated total cholesterol was reported in studies conducted in different parts of Ethiopia; Mekelle, 30.8% [18], Eastern Ethiopia, 33.7%, Hawassa, southern Ethiopia, 38.7% [16], and Togo, 64% [5]. In contrast, lower prevalence were reported in studies from Ethiopia, 5.2% [9], India, 15.3% [22], and Zambia, 15.8% [28].

The prevalence of elevated LDL-C in this study, 21.5% is comparable with the study findings reported in Hawassa, southern Ethiopia, 21% [16], Africa, 21.4% [4], India, 23% [22], and Venezuela, 23.3% [27]. However, it is higher than the study findings reported in China, 8.3% [1], Ethiopia, 14.1% [9], and India, 15.8% [29]. On the other hand, a higher prevalence of elevated LDL-C was found in Nigeria, 51%; similarly, it was reported to be the highest prevalent type of dyslipidemia in different parts of Ethiopia [6, 15, 18].

The prevalence of dyslipidemia in this study was significantly higher in those aged 30–39 years and ≥ 40 years, 68.5% and 87.4%, respectively, which is similar to the results reported in studies in Ethiopia, Iran, and China [1, 6, 15, 18, 23]. Gender was not significantly associated with dyslipidemia in this study. However, findings in many previous studies reported that women had a significantly higher prevalence of dyslipidemia [15–17, 26, 30]. Whereas, contradictory findings were reported in China and Saudi Arabia [1, 7]. The observed variations might be due to differences in gender composition, as in this study a relatively lower percentage of female participants were included. Additional reasons could be the diverse socio-economic status, culture, and lifestyle of the participants which could result in different dietary habits.

Alcohol does modulate several components of the lipogenic pathway; for instance, it decreases the primary enzyme that regulates lipogenesis resulting in higher levels of plasma lipids [31]. Similarly, several studies have reported that alcohol consumption is positively associated with dyslipidemia [1, 10–12, 32], which is consistent with the results of this study which revealed that alcohol consumption is significantly associated with dyslipidemia, mainly with reduced HDL-C and elevated triglycerides. This could be explained by working in an alcoholic beverage factory might increase the access to alcoholic beverages to the employees and the tendency of excessive alcohol consumption.

Similar to a previous study report in Eastern Ethiopia [6], less eating of vegetables is significantly associated with dyslipidemia in this study. The prevalence was higher among those who ate vegetables two days or less per week. Cigarette smoking was not significantly associated with dyslipidemia in this study, which is consistent with the findings reported in previous studies [1, 6, 26] and contrary to the finding reported in Saudi Arabia [7]. This variation might be due to differences in sample size and frequency of smokers among the participants.

In this study longer sitting duration was also significantly associated with dyslipidemia. Those who sat four hours or more per day had a higher risk of developing dyslipidemia. A comparable finding was found in India which reported a sedentary lifestyle, which could be described by longer sitting and lying down, was significantly associated with dyslipidemia [29]. This could be explained by longer sitting duration means fewer calories are being burned, which might lead to storage of calories in the form of lipids [33].

Raised waist circumference was also significantly associated with dyslipidemia in this study, which is consistent with the findings reported in various previous studies [1, 24, 26, 29, 34, 35]. This could be explained by as waist circumference increases energy might be stored in different forms of lipids. Contrary to the findings in many previous studies [9, 15, 16, 18, 28], BMI was not significantly associated with dyslipidemia in this study. This could be explained by the evidence that BMI was pointed as a poor indicator of obesity or body fatness in previous studies [34].

Studies conducted in India [22] and Durame, southern Ethiopia [15] reported dyslipidemia is significantly associated with hypertension. Other studies from Eastern Ethiopia and Saudi Arabia [6, 7] showed that elevated fasting blood sugar level was significantly associated with dyslipidemia. Whereas, this study reported a contradictory finding. This variation might be due to differences in study population and age composition. For instance, the study in Eastern Ethiopia was conducted solely in the female gender, the study in India includes only male participants above 30 years of age, and the study in Durame, southern Ethiopia was a hospital-based study. Besides, differences in socioeconomic status and lifestyle of the participants could also be additional reasons.

This study has several limitations. Firstly, as a cross-sectional study, it may not show the causal relationship between dyslipidemia and the assessed factors. Secondly, as an institution-based study, it lacks the ability to generalize the findings to the general population as a whole. Finally, using a purposive selection of the study site could have affected the representativeness of the sample. Despite these limitations, the findings of this study could be a great input to future broader studies.

## Conclusions

This study revealed a high prevalence of dyslipidemia among Awash wine factory employees in Addis Ababa. This finding indicates that dyslipidemia is a significant public health problem among the alcoholic beverage industrial working population of developing countries. Older age was the only non-modifiable risk factor for dyslipidemia in this study. Other modifiable factors such as; alcohol consumption, less eating of vegetables, longer sitting duration, and raised waist circumference were also the significant risk factors of dyslipidemia in this study. Periodic screening of high-risk groups for early detection and treatment is needed along with effective health promotion and education which encourages a healthy lifestyle. Furthermore, broader studies with different study designs are needed to assess the association of lipid derangements with alcohol consumption.

## Data Availability

The datasets used and/or analyzed during the current study are available from the corresponding author on reasonable request.

## Abbreviations

AIDS: Acquired Immune Deficiency Syndrome
AOR: Adjusted odds ratio
BMI: Body Mass Index
BP: Blood pressure
CHD: Coronary heart disease
CI: Confidence interval
COR: Crude odds ratio
CVD: Cardio vascular disease
DALYs: Disability adjusted life years
DBP: Diastolic blood pressure
DM: Diabetes mellitus
ETB: Ethiopian birr
HDL: High-density lipoprotein
HIV: Human immunodeficiency virus
IDF: International diabetic federation
IRB: Institution Review Board
LDL: Low-density lipoprotein
NCD: Non-communicable disease
NCEP-ATP: National Cholesterol Education Program-Adult Treatment Panel
OR: Odds ratio
SBP: Systolic blood pressure
SD: Standard deviation
SPSS: Statistical package for the social sciences
TC: Total cholesterol
TG: Triglyceride
WHO: World Health Organization
